# Investigating and improving pedestrian safety in an urban environment

**DOI:** 10.1186/2197-1714-1-11

**Published:** 2014-05-07

**Authors:** Keshia M Pollack, Andrea C Gielen, Mohd Nasir Mohd Ismail, Molly Mitzner, Michael Wu, Jonathan M Links

**Affiliations:** 1 Department of Health Policy and Management, Johns Hopkins Center for Injury Research and Policy, Johns Hopkins Bloomberg School of Public Health, Baltimore, MD 21205 USA; 2 Department of Health, Behavior and Society, Johns Hopkins Center for Injury Research and Policy, Johns Hopkins Bloomberg School of Public Health, Baltimore, MD 21205 USA; 3 Department of Health, Behavior and Society, Johns Hopkins Bloomberg School of Public Health, Baltimore, MD 21205 USA; 4 Johns Hopkins Bloomberg School of Public Health Diversity Summer Internship Program, Baltimore, MD 21205 USA; 5 Johns Hopkins University, Baltimore, MD 21205 USA; 6 Department of Environmental Health Sciences, Johns Hopkins Bloomberg School of Public Health, Baltimore, MD 21205 USA

**Keywords:** Pedestrian safety, Three E’s, Partnership, Urban environment

## Abstract

**Background:**

Prompted by a series of fatal and nonfatal pedestrian-vehicle collisions, university leadership from one urban institution collaborated with its academic injury research center to investigate traffic-related hazards facing pedestrians.

**Methods:**

This descriptive epidemiologic study used multiple data collection strategies to determine the burden of pedestrian injury in the target area. Data were collected in 2011 through a review of university crash reports from campus police; a systematic environmental audit and direct observations using a validated instrument and trained raters; and focus groups with faculty, students, and staff. Study findings were synthesized and evidence-informed recommendations were developed and disseminated to university leadership.

**Results:**

Crash reports provided some indication of the risks on the streets adjacent to the campus. The environmental audit identified a lack of signage posting the speed limit, faded crosswalks, issues with traffic light and walk sign synchronization, and limited formal pedestrian crossings, which led to jaywalking. Focus groups participants described dangerous locations and times, signal controls and signage, enforcement of traffic laws, use of cell phones and iPods, and awareness of pedestrian safety. Recommendations to improve pedestrian safety were developed in accordance with the three E’s of injury prevention (education, enforcement, and engineering), and along with plans for implementation and evaluation, were presented to university leadership.

**Conclusions:**

These results underscore the importance of using multiple methods to understand fully the problem, developing pragmatic recommendations that align with the three E’s of injury prevention, and collaborating with leadership who have the authority to implement recommended injury countermeasures. These lessons are relevant for the many colleges and universities in urban settings where a majority of travel to offices, classrooms, and surrounding amenities are by foot.

**Electronic supplementary material:**

The online version of this article (doi:10.1186/2197-1714-1-11) contains supplementary material, which is available to authorized users.

## Background

In fall 2011, approximately 76 million people were enrolled in U.S. schools and colleges, and nearly 6 million professional, administrative, and support staff worked at educational institutions (Snyder and Dillow 2012). Many of these postsecondary education institutions do not allow students to possess cars; for others, it is impractical because of limited roadway access or parking. Thus, the majority of students’ mode of transportation to and from campus and surrounding amenities is primarily as pedestrians. The resulting scenario is one where students may be at increased risk of pedestrian injury when traveling in areas adjacent to campus. This risk is heightened for individuals attending school or working at campuses located in urban areas with high traffic volume.

In 2010, over 70,000 pedestrians were injured and 4,200 pedestrians were killed in traffic crashes in the U.S. (fatalities accounted for 13% of total fatalities due to traffic) (NHTSA 2012). Nearly 75% of these pedestrian fatalities occurred in urban environments (NHTSA 2012). Prior research has identified young and old age, and consumption of alcohol, as some of the strongest individual level factors (Dutz et al. 2011; Schwebel et al. 2012). Certain driver behaviors, such as speeding, and environmental factors such as inadequate vehicle design are also correlated with increased risk of pedestrian injury (Han et al. 2012; DiMaggio and Li 2012).

Pedestrians also engage in risky behaviors in terms of distracted walking. Hyman et al. (2010) found that pedestrians using cell phones took a longer time to get to their destination, changed directions frequently, and were less observant of unusual surroundings at considerably higher levels than those pedestrians who were not on their cell phones. Pedestrians talking on cell phones in a simulated environment took longer to cross the street and paid less attention to traffic; overall, they had more missed opportunities crossing against traffic and more simulated hits or close calls than those pedestrian with no cell phone distraction (Stavrinos et al. 2011; Schwebel et al. 2012).

College students may be a particularly vulnerable as pedestrians, because their mode of transportation is typically on foot and many may be coming to a new area with an unfamiliar pedestrian environment. Few studies in the peer review literature have explored the risks that college students face as pedestrians. Wojtowicz and DesLauriers’ (1996) analyzed fifteen campus crosswalks at a single campus and showed a direct relationship between risk of collision and high traffic intersections. Not surprisingly, the study indicated that the higher traffic intersections had a greater risk of pedestrian-vehicle collision. This study and a more recent one by Schneider et al. (2004) also emphasized the importance of the built environment, including non-continuous sidewalks and crosswalk density, in contributing to pedestrian crashes in the streets adjacent to a college campus. In addition, students may be at risk of sustaining a pedestrian injury because of heavy reliance on technology and increased attention on “distracted pedestrians” (Byington and Schwebel, 2013; Nasar and Troyer, 2013).

Over a two-year period at one undergraduate institution in an urban environment, several fatal and nonfatal incidents involving students occurred while they were walking near the campus (Schellenbach 2012). A review of these crashes suggested one primary area of concern for pedestrians, and a perception that the incidents were due to a combination of speeding and illegal street crossing by pedestrians. These crashes prompted university leadership to collaborate with its academic injury research center to explore ways to understand traffic-related hazards and mitigate pedestrian injury risks facing students, staff, and employees. This article documents the comprehensive approach the university undertook to understand and address this public health problem. To inform the development of a comprehensive intervention to improve pedestrian safety, and address the scant knowledge in this area, leadership at one urban institution partnered with its academic injury research center to: 1) conduct an environmental audit of the high-crash risk areas; 2) understand how students, faculty, and staff think and talk about pedestrian and bicycle safety; 3) obtain a general sense of the typical walking patterns and behaviors of students, faculty, and staff; and 4) make recommendations for improving pedestrian safety in and around the campus. The purpose of this article is to describe the results of this investigation, make research-informed recommendations to improve pedestrian safety, and discuss the implications for other institutions facing similar challenges.

## Methods

The approach taken to investigate the problem of pedestrian safety in the target areas involved a descriptive epidemiologic study with data collection via multiple methods. The framework of the “three E’s”, education, enforcement, and engineering, guided our methods. We relied on collecting data within the scope of the three E’s because interventions that include all three components have effectively improved pedestrian safety in other settings (Staunton et al. 2007; Boye and Geller, 2000; Standfast, 1988). The focus of the activities described here was the Johns Hopkins University Arts and Sciences campus, which includes mainly undergraduate programs. The average total undergraduate enrollment is about 5,200, and the campus size is 140 park-like acres. The University is located in Baltimore and is bordered by several of the City’s thoroughfares running both North–south on the east side of campus, and East–west on both the North and South sides of campus.

Data collection occurred through a review of existing university crash data, an environmental audit and direct observations, and focus groups with faculty, students, and staff. A review of the literature was also conducted to inform development of the focus group guide and identify appropriate tools for the environmental audit. An Ad Hoc Pedestrian Safety Committee comprised of university leadership, students, and the injury researchers who led this project, helped to guide the scope of the project. The Johns Hopkins Bloomberg School of Public Health Institutional Review Board approved these study procedures.

### Review of university crash data

Due to the urgency of the issue, reports on all crashes that occurred from 1/1/2009-6/1/2011 were collected from the University’s Security Department. These years of data were selected to correspond with the period when most of the crashes occurred, and in turn prompted university action. A total of n = 59 crashes were reported during the study period, and reports were available for each of these incidents. Campus police use a standard form to record a description of each crash, which for each case listed the location, occurrence date and time, other notable characteristics, and a narrative. The descriptive fields and narratives were reviewed and summarized accordingly. These data were used to identify high-crash areas.

### Environmental audit

**Figure 1:**
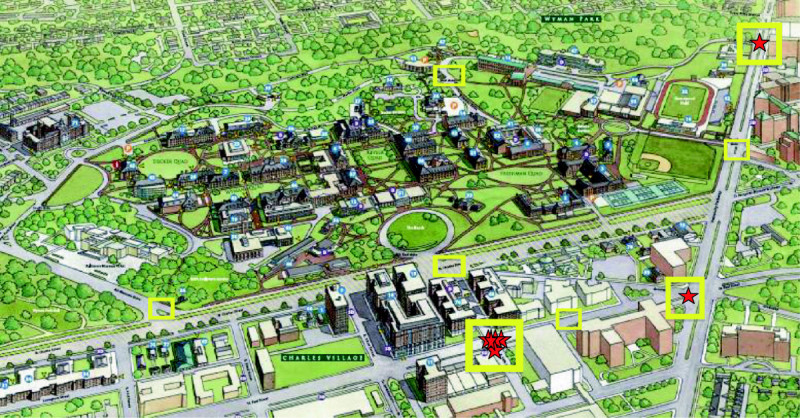
**University Campus**^**1,2**^ **.** ^1^Red Star – location of pedestrian-vehicle collisions. ^2^Yellow squares indicate key areas of more than one traffic-related environmental feature of concern (lack of signage, faded crosswalks, etc.).

The pedestrian-vehicle collisions that led to this study all occurred at intersections surrounding the campus. Thus, the team began by visiting these intersections to provide a better understanding of the situation, and identify areas for the environmental audit. Next, with input from the University’s Ad Hoc Committee studying this issue, 25 roads segments around the perimeter of the campus were selected for the environmental audit. These roads were perceived as the ones that had the most pedestrian traffic, and thus where a collision would likely occur. The Appendix includes a map (Figure 1) illustrating these segments and locations of the prior incidents.

The Walking Suitability Assessment Form V.021003 (WABSA), a one-page valid instrument designed to collect less than a dozen key characteristics of any road segment (e.g., sidewalk width number of through lanes, posted speed limit) was used for data collection (Emery et al. 2003; Emery and Crump, 2003; Emery 2003). This observational audit instrument was developed to assess objective data on walking suitability. The WABSA is intended for use by laypersons to collect systematic data to identify problems in the built environment (Emery 2003). The criterion-related validity has been reported at 0.58 and intercoder reliability at 0.79. The validity and reliability correlations for individual items are reported elsewhere (Emery et al. 2003). The validity of the tool is strongest when assessing streets within urban and suburban areas (Hansen et al. 2009; Seagle et al. 2008), as was the case here.

One of the study’s co-principal investigators, with experience measuring the built environment, led this component of the project and trained three research assistants in using the WABSA. During the training, the co-investigator and raters independently rated several block segments, compared their findings, and discussed discrepancies. After each rater was trained, the 25 road segments adjacent to the campus were divided among the raters. Each rater was also provided with a camera and a tape measure, in order to take photos of hazards and to measure sidewalk widths. The audit tool specifically measured the walking environment and included the posted speed limit, number of lanes, presence of a sidewalk, sidewalk material, condition and width; buffer width; curb ramps; street lights; intersections; and isolated problem spots. The instrument also had specific questions about the nearby intersections. Finally, the instrument included a field for the coder to document notable pedestrian behaviors (e.g., jaywalking, person crossing while on the phone). As part of the training, the raters learned about possible options for this open-ended field.

Assessments were completed over the span of a 4-week period during the daylight hours only. Since the raters individually collected data, the audits were completed in the daytime to address security concerns, and to coincide with the times of the day when the greatest number of pedestrians were in the target areas (morning and late afternoon rush hour periods). Once data collection was complete, the lead investigator on this component of the study enumerated the road segments, randomly selected 25%, and re-assessed the segments to validate the initial measures. Data from the assessment forms were used t generated a walkability score was calculated for each segment. These scores were categorized into an ordinal scale of walkability (unsuitable, very poor, poor, fair, good, to very good) according to the guidebook (Emery et al. 2003).

### Focus groups

During the summer months of 2011, two focus groups were conducted, one with students and another with faculty and staff, to better understand pedestrian safety perceptions and typical patterns, as well as identify risky areas and ideas for improving pedestrian safety. Students, staff, and faculty were the populations targeted for these focus groups; collecting community perspectives was not part of the initial scope of work. Students were recruited via an email sent by the Office of the Dean of Students, and by flyers distributed by student research assistants. Faculty and staff were recruited in two ways: (1) those who had contacted the project when they read about our work in one of the university publications were invited by email to attend; and (2) the Ad Hoc Pedestrian Safety Committee identified key individuals who had knowledge and experience with the subject who were also invited by email.

The student focus group occurred in the evening, and the faculty and staff focus group in the morning, and a light meal was provided for each. Oral informed consent was collected prior to beginning each focus group, which lasted from 60–90 minutes. Two members of the study team, experienced with qualitative methods, facilitated the discussions, with the student research assistants serving as note takers. Audiotapes were transcribed and a verified by one of the student research assistants. Open coding was used to analyze the data (Saldana 2013). This process involved the two investigators, and the two research assistants who had attended the sessions, reading all of the transcripts to determine dominant themes and recommendations for improvement that emerged across the focus groups. The two research assistants individually prepared written summaries of their conclusions, and the four-team members met to discuss the various perspectives and findings. Based on this discussion, the summaries, and the written transcripts, one of the study’s co-principal investigators then synthesized the results into a final set of themes and recommendations. This synthesis was shared with all four team members for final comments and consensus was reached.

## Results

### Identifying high-risk areas

Fifty-nine crashes were reported to the university Security Department on main city streets adjacent to the campus during the study period. A total of 12% (n = 7) involved a pedestrian; the remaining incidents involved a single or multi-vehicle crash. These seven incidents involving pedestrians occurred mainly between 6:00 pm and midnight and between noon and 6 pm. Four of these incidents occurred when the weather was clear. According to the narratives, the most common injury circumstance involved the driver turning or failing to stop at a red light or stop sign.

Assessments were conducted on 25 street segments (including intersections), most of which were rated as fair or poor. Several intersections were noted as especially busy and lacking synchronization between the “walk” signal and the traffic lights. Key findings of the audit indicated: a lack of signage posting the speed limit; faded crosswalks; issues with traffic light and walk sign synchronization (timing is such that in many instances pedestrians are provided the walk signal at the same times that cars are permitted to turn); and limited formal pedestrian crossings, which led to unsafe crossing (i.e., significant jaywalking). In addition, observations of crossing behavior that were noted while conducting these audits revealed long durations of time that pedestrians spent prior to crossing, which perpetuated unsafe crossing, especially when pedestrians did not have the right of way to cross the street. In addition, several individuals (appeared to be students) were seen crossing the street while wearing headphones or using their handheld device (especially a cell phone or smart phone or iPod) while vehicles appeared to be traveling at high speeds.

### Safety perceptions and behaviors

The focus groups provided additional data to further investigate this issue. A description of the 15 individuals (7 students and 8 faculty/staff) who participated in the focus groups is summarized in Table 1. Across the two focus groups, five themes emerged: 1) dangerous locations and times; 2) signal controls and signage; 3) enforcement of traffic laws; 4) use of cell phones and iPods; and 5) awareness of pedestrian safety. Intersections that were perceived as having the most pedestrian traffic were viewed as the most risky. Comments centered on the speed that cars traveling through those intersections. In addition, the uneven sidewalks on certain street segments were also mentioned, which made walking treacherous, particularly during inclement weather. All of the focus group participants noted that one particular street that lacked sidewalks, which made travel very dangerous. The most dangerous times according to students were early in the morning when students were focused on getting to an 8:00 am class and late at night:

“And I see people run lights, red lights at night but this is Johns Hopkins University. People are up 24/7… and so if I'm walking around at night time and I see people doing close to 50 miles up Charles Street.”

**Table 1:** **Demographic characteristics of focus group participants (N = 15)**

	Student focus group (n = 7)	Faculty/Staff focus group (n = 8)
Age (years)		
19	5	0
20-29	2	1
30-49	0	2
50+	0	5
Gender:		
Male	1	5
Female	6	3
Year in School:		
Junior	6	N/A
Senior	1	
Years employed at University		
0-5	N/A	1
6-10		5
11+		2
Primary Mode of Transportation		
Walk	6	3
Car	1	2
Bike	0	3
Average Time Spent Walking on Campus		
≤ 4 hours per day	4	8
≥ 5 hours per day	3	0

Both focus groups talked about the beginning of the academic school year as a particularly dangerous time because of the influx of students, many of whom are from other places that likely have very different traffic and pedestrian environments.

A consistent finding across the two focus groups was that a large part of the problem with dangerous intersections had to do with the signal controls that allow vehicles to turn at the same time that pedestrians have the walk signal. For instance, in describing a particular location, one student noted:

“Some traffic lights which are not really in synch for people as in they stay at green light until you cross the street but cars are also turning as well so you try to go across and can't, like “Hey I want to cross,” but the cars are turning as well so I can't really cross.”

A participant in the faculty/staff focus group describes a similar situation at another intersection on the same street:

“I still think this light here…[cars] get that green arrow to turn right, but I also think at the same time, the pedestrians are getting the walk signal. So you’re basically telling people to walk, and you’re telling people to make that right turn at the same time. And I’m like, “Are they gonna stop?”

Students and faculty both described safer pedestrian environments that they had encountered in other cities where the traffic stops in all directions to allow pedestrians to cross in any direction – a so-called “pedestrian scramble.” With regard to signage, both faculty/staff and student focus groups reported that signage was minimal, confusing, or ignored.

The general sense among the students was that drivers in the city do not pay attention to traffic rules such as speeding and pedestrian right of way. Perhaps because of this concern about drivers and the problems with intersection crossing signals, students generally found it acceptable, and sometimes safer (as perceived), to jaywalk. As one student pointed out, “it's safer to look for traffic instead of following the signs.” Another student said:

“I'm not from the city. I'm from the suburb area, but around here sometimes jay walking is the safest way because try to follow pedestrian things and nobody wants to listen…I mean I haven't got stopped by the police but I think most of my times here I jaywalk and that's just because I'm out in the open.”

Students in the focus groups uniformly agreed that walking while talking or texting on a cell phone, or listening to music on an iPod, was a common practice. Although they did comment that they had seen instances when a pedestrian was “oblivious” to impending danger because they were listening to their iPod or on the phone, they did not think it was a particularly dangerous practice. The students felt that they are keenly aware of when they need to pay attention to the traffic. For instance, they reported that they would stop texting when they reached an intersection, or they would turn the music down when they thought they needed to be able to hear.

Both focus groups discussed the general lack of information about pedestrian safety available on campus and offered suggestions as to how to increase awareness. Orientation and weekly campus tours were suggested as options to incorporate information. The students underscored the urgency of doing something in response to the recent incidents. Students said that messages should address the belief that “it can’t happen to me” and that they would prefer messages delivered by outside experts who could share the data.

Although the faculty/staff focus group participants agreed that raising awareness was important, one participant pointed out that:

“It’s also really hard to do an education campaign because there’s a total complete turnover every four years. And so unless you’re really committed to doing this all the time, and the costs really will be substantial, it’s almost useless.”

An alternative focus on drivers was suggested by a participant:

“Although there is turnover students every four years, whatever, I suspect a lot of these drivers are driving this route every day for much longer than four years. And that we could acclimate them to there being pedestrians…I mean, I don’t know that that’s possible, but I suspect that these are commuters who go the same route every day. We could teach them.”

Increasing awareness of pedestrian safety was thought to be necessary but probably insufficient to address the problem. They also thought improved markings on roadways (as is the case in public school zones) and increased lighting at night at high volume pedestrian and motor vehicle traffic locations would help. Overall, the students were not supportive of ideas to modify the environment by creating roundabouts or speed bumps; however, they were quick to point out that decisions about such changes needed to be based on more research specific to the campus situation. Students and faculty both discussed the potential benefits of having individuals “on the street” to help with promoting pedestrian safety, especially at high-risk times. Faculty and staff talked about having more crossing guards available to assist pedestrians when crossing the street.

### Developing recommendations for prevention

Data collected from each aspect of this study (university crash reports, audit, literature review, and focus groups) were synthesized to generate conclusions and recommendations. Drawing on the data, and nationally recognized guidelines for pedestrian safety (Redmon 2011), several recommendations consistent with the three E’s were generated. These recommendations are summarized in Table 2. They were shared with the university’s Ad Hoc Pedestrian Safety Committee as a final report and PowerPoint slide presentation.

**Table 2:** **Recommendations to improve pedestrian safety, by the three E’s (Education, Engineering, Enforcement)**

**Education**	• During new student orientation, as part of one of the mandatory sessions, include sessions on pedestrian safety.
	• Include additional information on pedestrian safety during the police walks that occur for all freshmen that opt to do this.
	• Develop a communications campaign on pedestrian risks to improve awareness and knowledge about how to travel safely as a pedestrian.
	• Obtain and utilize input from students to plan all educational and communication messages and materials.
**Enforcement**	• Utilize crossing guards at high traffic intersections.
	• Increase traffic law enforcement, especially at the beginning of the school year.
	• Encourage law enforcement to ticket pedestrians for jaywalking, which could initially be giving out warnings, but with the potential for tickets with a fine.
	• Install speed cameras to help slow down the traffic. Direct observations noted that many vehicles traveled at speeds in excess of posted speed limits.
	• Install red light cameras. A review of the crash reports noted instances when vehicles were making illegal turns.
**Engineering**	• Add in-street pedestrian crossing signs to remind drivers to stop for pedestrians.
	• Post additional speed signage for vehicles. Currently few signs are located on high-traffic roads.
	• Changes to traffic light and walk sign synchronization (i.e., so pedestrians can’t be struck by turning traffic), especially for those turning. Consider installing a delay for cars to allow pedestrians to safely cross before the vehicle.
	• Reduce wait time at lights (consider having push buttons that pedestrians can activate to request the walk signal)
	• Having lights with the countdown function that lasts for the duration of the pedestrian crossing, for instance, count down the full 60 seconds that pedestrians have to cross, may be a better technology to implement at various crosswalks.
	• Consider installing speed humps or other traffic calming devices at various locations to slow traffic.

## Discussion

Although discussion about safe routes to schools target elementary and middle school students, pedestrian safety is also an important public health problem for students and employees on university and college campuses. Surprisingly, there were no studies in the peer review literature that evaluated and documented interventions on university campuses. Furthermore, of the limited peer review literature that described pedestrian safety in this population, none that we identified included understanding students and staff perceptions on pedestrian safety (Wojtowicz and DesLauriers 1996; Schneider et al. 2004). Of all of the data collected for our present research, we found the information collected during the focus groups particularly important when developing recommendations. Although these data resulted from a single case, our review of the peer-reviewed and grey literature revealed several media reports of individuals who were involved in a pedestrian-vehicle collision near a university campus. Other institutions seeking to reduce pedestrian injury risk could replicate the approach used here. This research also illustrated the importance of pedestrian safety due to traffic, as well as crime, for urban institutions, which unintentionally may be overlooked.

Framing the study and recommendations around the three E’s was a strategy that worked particularly well, especially for the non public health stakeholders involved in this initiative. Doing so also helped organize the recommendations, so that priorities could be identified within each of the three E’s. Regarding education, some strategies were identified to address the lack of awareness regarding pedestrian safety. There was a sense from the focus group participants and the Advisory Committee that some students and staff were unfamiliar with urban environments. The University focused quite a bit on pedestrian safety in regards to crime, and did not include traffic-related hazards. Thus, it was suggested that education about pedestrian risk be expanded to include crime as well as traffic. This recommendation was adopted, and included in the 2012 and 2013 new student orientation. In addition, the university sponsors an optional safety walk around the campus for all first-year undergraduate students, and in direct response to this research, this walk was expanded to cover both crime and traffic risks. The results from this study also in part informed the development of an education campaign, Road Scholar, led by the University’s Office of Communications with input and participation by the students. The focus group transcripts were shared with the campaign developers, along with additional data collection from the students specifically regarding the campaign. This campaign used messaging and signage to remind pedestrians to be cautious and alert of their environment, in essence to create road scholars. An article describing the campaign was published in the institution’s magazine (Schellenbach 2012).

For enforcement, recommendations were generated from the focus groups that related to the use of crossing guards and technology such as speed cameras. The University hired local enforcement to provide navigate traffic and pedestrians at the key intersection where most of the pedestrian-vehicle collisions occurred. This increased enforcement also helped to reduce the number of illegal turns that were being made at the red light at this intersection (there was a ‘no turn on red’ sign at the intersection); illegal turns were mentioned in several of the crash reports. The observations noted while conducting the environmental audit revealed several instances where vehicles traveled at speeds in excess of the posted speed limit on the main corridor near the University (where most incidents occurred). As a result, University leadership advocated for a speed camera to help slow down traffic along the main corridor.

The audit also identified potential environmental changes that could be implemented, including adding additional signage and a dedicated turning arrow at the most hazardous intersection (Table 2). The University’s leadership considered the environmental changes and some of them were included in a request to the City for ways to make the neighborhoods adjacent to the campus safer for pedestrians. The City is in the process of redeveloping the area near the campus, and it is possible that some of the engineering recommendations could be incorporated to the project.

### Study limitations

While we utilized a systematic and rigorous approach to collecting data, due to the urgency to develop solutions that could be implemented at the beginning of the next academic year, data collection for this case occurred in the summer months. Although a good proportion of the student body was present over the summer, the volume of pedestrian traffic was lower than during the school year. In addition, vehicular traffic flow may have also been lower than usual, because travel patterns and work schedules may vary during the summer. Thus, the results may not be generalizable to the rest of the year.

In addition, this research relied on University crash data and did not collect data from the police department. At the inception of this study, due to the quick timeline to complete the research data were only collected from the University security office. While all of the available reports were reviewed, these data are biased in that they capture only those who are affiliated with the institution and who reported the incident. Incidents only reported to the local police department were not included in these data, thus these data may not be generalizable to the larger community. While we sought to recruit widely for the focus groups, the sample may have been biased in that individuals who wanted to express their concerns regarding pedestrian safety were more likely participate. However, by speaking with students, faculty, and staff, we feel that we did capture a broad range of responses that were raised at both focus groups. Moreover, due to limited resources, student and faculty/staff input was limited to two focus groups, so our results do not reflect the broader student/faculty/staff or the neighboring community’s perspectives. In regards to this issue, since the University community liaison was part of the Ad Hoc Committee, some of the community concerns on this issue, which had been raised at local neighborhood association meetings, were also raised during the Committee meetings.

## Conclusions

Pedestrian safety remains an important public health problem, and effective evidence-based solutions exist. This case highlights how one university located in an urban environment brought together researchers and leadership to measure and better understand specific risk to pedestrians, garner their thoughts on solutions, and prioritize strategies to implement. Although this article documents the experience of a single institution the information presented is relevant for similar settings globally, especially where academic institutions are in urban environments, and pedestrian risks to students and employees are great. Moreover, the array of methods used in this descriptive epidemiologic study also provides important guidance for others in the field seeking to conduct a similar investigation to understand the problem and determine appropriate solutions. While the recommendations were collected around the three Es, it is important to note also that the research team heavily emphasized the fourth E, evaluation. Thus, the study team is planning to assess which interventions were implemented, and continue to monitor pedestrian-vehicle collisions to determine if these efforts resulted in a safer urban environment for pedestrians. Based on the work accomplished in this case, the injury research center is currently employing similar methods at one of the university’s other campuses.

## Authors’ original submitted files for images

Below are the links to the authors’ original submitted files for images. Authors’ original file for figure 1
