# Supplementary figures and images for: Investigating and improving pedestrian safety in an urban environment
Source: Inj Epidemiol. 2014 May 7;1(1):11. doi: 10.1186/2197-1714-1-11 (PMC5005641; doi:10.1186/2197-1714-1-11)

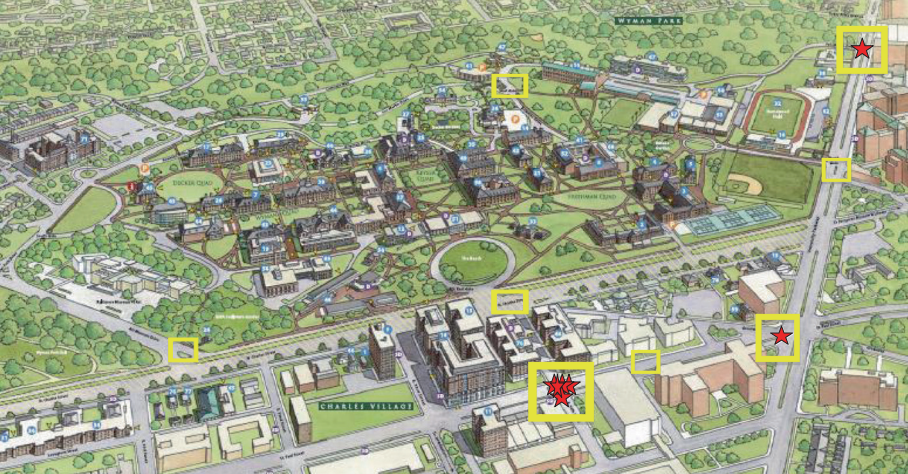

Supplement: Supplementary file 1 — Authors’ original file for figure 1 [file 40621_2013_11_MOESM1_ESM.pdf]
